# Exercise-Training Regulates Apolipoprotein B in *Drosophila* to Improve HFD-Mediated Cardiac Function Damage and Low Exercise Capacity

**DOI:** 10.3389/fphys.2021.650959

**Published:** 2021-07-07

**Authors:** Meng Ding, Lan Zheng, Qiu Fang Li, Wan Li Wang, Wan Da Peng, Meng Zhou

**Affiliations:** Key Laboratory of Physical Fitness and Exercise Rehabilitation of Hunan Province, Hunan Normal University, Changsha, China

**Keywords:** apolipoprotein B, exercise-training, cardiac dysfunction, lipid metabolism, *Drosophila*, exercise capacity

## Abstract

Apolipoprotein B plays an essential role in systemic lipid metabolism, and it is closely related to cardiovascular diseases. Exercise-training can regulate systemic lipid metabolism, improve heart function, and improve exercise capacity, but the molecular mechanisms involved are poorly understood. We used a *Drosophila* model to demonstrate that exercise-training regulates the expression of *apoLpp* (a homolog of apolipoprotein B) in cardiomyocytes, thereby resisting heart insufficiency and low exercise capacity caused by obesity. The *apoLpp* is an essential lipid carrier produced in the heart and fat body of *Drosophila*. In a *Drosophila* genetic screen, low expression of *apoLpp* reduced obesity and cardiac dysfunction induced by a high-fat diet (HFD). Cardiac-specific inhibition indicated that reducing *apoLpp* in the heart during HFD reduced the triglyceride content of the whole-body and reduced heart function damage caused by HFD. In exercise-trained flies, the result was similar to the knockdown effect of *apoLpp*. Therefore, the inhibition of apoLpp plays an important role in HFD-induced cardiac function impairment and low exercise capacity. Although the *apoLpp* knockdown of cardiomyocytes alleviated damage to heart function, it did not reduce the arrhythmia and low exercise capacity caused by HFD. Exercise-training can improve this condition more effectively, and the possible reason for this difference is that exercise-training regulates climbing ability in ways to promote metabolism. Exercise-training during HFD feeding can down-regulate the expression of *apoLpp*, reduce the whole-body TG levels, improve cardiac recovery, and improve exercise capacity. Exercise-training can downregulate the expression of *apoLpp* in cardiomyocytes to resist cardiac function damage and low exercise capacity caused by HFD. The results revealed the relationship between exercise-training and *apoLpp* and their essential roles in regulating heart function and climbing ability.

## Introduction

Obesity is caused by the imbalance between energy input and output and the interaction between environmental factors and susceptibility genes (Barsh et al., 2000). Obesity is the most common polygenic disease and it is closely related to cardiovascular disease (Russo et al., 2010). Obesity increases the risk of cardiovascular disease, increases blood lipids, and impairs exercise capacity (Van Gaal et al., 2006). Severe obesity is closely related to the incidence of cardiovascular disease (Ortega et al., 2016). Diet is an essential factor affecting obesity (Hill et al., 2000; Heinrichsen et al., 2013). The lipids in food (mainly triglycerides, phospholipids, and cholesterol esters) are hydrolyzed in the intestinal lumen and absorbed by the intestinal cells. Lipids are re-synthesized in the endoplasmic reticulum (ER) and then packaged into lipoprotein particles (chyle microparticles, or CMs) or cellular lipid droplets for long-term storage (Kühnlein, 2011). The surface of CMs are covered with a monolayer of phospholipids, containing free cholesterol and apolipoprotein, which play an essential role in the transportation of triglycerides and fat-soluble vitamins. The assembly of CMs relies on apolipoprotein B (apoB) and microsomal triglyceride transfer protein (MTP). When CMs mature, they can transport lipids to the required tissues to provide energy and hydrolyze in the circulation; their products are absorbed by peripheral tissues (Hussain, 2014; Heier and Kühnlein, 2018). For humans, obesity leads to an increased risk of cardiovascular disease (Zhang and Ren, 2016; Wu et al., 2019). Exercise-training (E) can alleviate this adverse effect (Klein et al., 2004; Swift et al., 2014). In mice and rats, exercise-training can alter the metabolic abnormalities caused by a high-fat diet (HFD). The absence of apolipoprotein B produces decreased plasma TG and cholesterol levels (Watts et al., 2009; Miotto et al., 2017). In addition, exercise training can reduce the increase in apolipoprotein B induced by HFD (Holme et al., 2007; Rosenkilde et al., 2018).

*Drosophila* is an excellent model for studying cardiovascular function. In *Drosophila* and mammals, most of the genes that regulate cardiac differentiation are conserved and the genetic regulation of cardiac function may also be conserved (Ocorr et al., 2007). The major lipoproteins of *Drosophila* and other insects, the lipophorins (Lpp), are similar to mammalian apoB-containing lipoproteins; their scaffolding apolipoproteins, the apolipophorins (*apoLpp*), are members of the apoB family, which is conserved throughout the animal kingdom (Palm et al., 2012). The *apoLpp* of the apolipoprotein B family is the primary hemolymph lipid carrier. Lipoprotein transports neutral lipids, phospholipids, and sterols between different organs and is associated with lipid metabolism diseases. When a certain amount of HFD is consumed, the lipids are hydrolyzed in the intestine. The product is absorbed by the intestinal cells and then re-synthesized and packaged in the endoplasmic reticulum. Then lipoprotein Lpp is recruited to the intestine, combined with lipid particles and transported to other tissues for use (Palm et al., 2012). The heart regulates systemic lipid metabolism and maintains lipid homeostasis. On a HFD, the cardiomyocyte-derived apoB-lipoproteins are the predominant determinants of whole-body lipid metabolism (Lee et al., 2017).

Exercise-training can improve heart function and reduce type 2 diabetes and cardiovascular disease (Martinez-Gomez et al., 2019). *Drosophila* has been used as a model to study the mechanisms and methods of heart function (Diop and Bodmer, 2012). An algorithm for the *Drosophila* heartbeat can be used to accurately analyze heart rate, heart period, systolic interval, diastolic interval, arrhythmia index, diastolic diameter, systolic diameter, fibrillations, and fractional shortening (Fink et al., 2009). Taking advantage of the rapid iterative negative geotaxis (RING) (Gargano et al., 2005) in *Drosophila*, we developed fly exercise devices, exercise-training, and designed exercise programs (Zheng et al., 2015). Appropriate exercise has been proven to prevent adverse changes in the structure and function of the heart (Ascensão et al., 2007; Marzetti et al., 2017; Cattadori et al., 2018), but its molecular mechanism is poorly understood. Therefore, our purpose is to study the relationship between exercise-training and the expression level of *apoLpp*, and their role in cardiac function and exercise capacity.

## Materials and Methods

### Fly Stocks and Groups

The w^1118^ (Wild-type fly) and *Hand-Gal4* (cardiomyocyte-specific drive) were obtained from Xiu-shan Wu (Heart Development Center of Hunan Normal University). UAS-*apoLpp*^RNAi^ (y1 sc^∗^ v1 sev21; P{TRiP.HMS00265}attP2/TM3, Sb1) flies were obtained from the Bloomington Stock Center. Through the hybridization of *Hand-Gal4* or W^1118^ with UAS-*apoLpp*^RNAi,^ flies with *apoLpp* knockdown of the cardiomyocytes or the whole-body were produced (Lee et al., 2017). In addition, for the convenience of writing. We define a high-fat diet as HFD, a normal diet as NFD, and exercise-training as E.

UAS-*apoLpp*^RNAi^ female flies were crossed with W^1118^ male flies and female flies whose F1 generation eclosion was collected within 12 h. These flies were defined as control groups, and different interventions were applied. That is NFD + W^1118^ > *apoLpp*^RNAi^, NFD + E + W^1118^ > *apoLpp*^RNAi^, HFD + W^1118^ > *apoLpp*^RNAi^, HFD + E + W^1118^ > *apoLpp*^RNAi^. UAS-*apoLpp*^RNAi^ female flies were crossed with *Hand-Gal4* male flies and female flies whose F1 generation eclosion was collected within 12 h. These flies were defined as cardiomyocyte-specific knockdown of apoLpp groups, and different interventions were applied. That is NFD + Hand-Gal4 > *apoLpp*^RNAi^, NFD + E + Hand-Gal4 > *apoLpp*^RNAi^, and HFD + Hand-Gal4 > *apoLpp*^RNAi^, HFD + E + Hand-Gal4 > *apoLpp*^RNAi^. UAS-*apoLpp*^RNAi^ female flies were crossed with *Arm-Gal4* male flies and female flies whose F1 generation eclosion was collected within 12 h. These flies were defined as whole-body apoLpp knockdown groups, and different interventions were applied. That is NFD + Arm-Gal4 > *apoLpp*^RNAi^, NFD + E + Arm-Gal4 > *apoLpp*^RNAi^, HFD + Arm-Gal4 > *apoLpp*^RNAi^, HFD + E + Arm-Gal4 > *apoLpp*^RNAi^. All flies were placed in transparent glass tubes, 20–30 per tube. NFD-fed flies were placed in a constant temperature and humidity incubator (25°C, 50% humidity, 12-h day-night cycle). HFD-fed flies needed to be reared in an environment of 21–22°C to prevent part of the high-fat food from melting and causing the flies to stick to the food and die.

### Diet Preparation

The preparation of the standard medium is as before (Birse et al., 2010; Wen et al., 2017). The high-fat medium was made by mixing 30% coconut oil was added to the food in a weight to volume ratio with the standard medium.

### Exercise-Training Device and Protocols

We developed a *Drosophila* movement device, which accounts for *Drosophila*’s negative tropism climbing movement by controlling the glass tube’s overturn. Each vial was rotated along its long axis with the accompanying rotating steel tube, which made the flies climb. Most flies continued to respond by climbing throughout the exercise period. The few that failed to climb were actively walking along the inner wall of the vial (Zheng et al., 2015; Wen et al., 2017). The distance between the glass tube and the sponge plug was adjusted before exercise so that the flies had 8 cm of space to complete the exercise. In addition, the temperature controller, humidifier, and electric switch were used to simulate the environment in the incubator so that the environment in which the flies exercise was similar to the incubator. Before the flies received exercise-training, all flies were fed under NFD conditions for 5 days. The flies in the exercise-training group started training on the 6th day and lasted for 5 days, and ended the training on the 10th day of age. The *Drosophila* exercise device’s flip frequency was 24 s/revolution, and the flies were exercised for 1.5 h a day for 5 days. Note: When doing exercise-training, the flies were first transferred from the glass tube with medium to the empty glass tube. After the exercise was completed, the flies were transferred to previous medium.

### Feeding Protocol

Parental flies harboring the appropriate Gal4 driver with parental flies harboring UAS-transgene ^RNAi^ for Gal4/UAS crossing. The parental female and male flies were usually loaded into fresh NFD medium after 2–3 days to expand the culture (2–3 days are sufficient to deposit a layer of eggs on the surface of food). The offspring flies emerge at approximately 12 days, and then the female flies that emerge within 12 h are collected. All fruit flies needed to be fed on normal medium for 5 days first and then transferred to fresh normal medium or the high-fat medium on the 6th day for another 5 days. The heart was collected or the whole flies were dissected on flies 11 days old. Note: High-fat medium needed to be placed at 21–22°C, and normal medium at 25°C.

### Real-Time Quantitative PCR (qPCR)

60 hearts or 10 whole-body tissues were put into 1 mL of Trizol reagent lysis solution for homogenization and extract RNA. Trizol extracted the organic solvent, and 10 μg total RNA was purified using oligo (dT) were synthesized from total RNA with superscript II reverse transcriptase (Invitrogen). qPCR amplification reactions were performed in triplicate, by mixing 1 μl of RT product with 10 μl of SYBR qPCR Mastermix (TaKaRa) containing the appropriate PCR primers. Thermal cycling and florescence monitoring were performed in an ABI7300 (Applied Biosystems, United States) using the following PCR conditions: (30 s at 95∘C, 5 s at 95∘C, 30 s at 60∘C) × 40. Values were normalized with gapdh. Primers used were as follows:

Gapdh F: 5′-GCGTCACCTGAAGATCCCAT -3′
R: 5′-GAAGTGGTTCGCCTGGAAGA -3′
*apoLpp* F: 5′-AATTCGCGGATGGTCTGTGT -3′
R: 5′-GCCCCTTAGGGATAGCCTTT -3′

### Semi-intact *Drosophila* Heart Preparation and Heartbeat Analysis

Based on previous research methods (Fink et al., 2009). First, 30 flies were anesthetized with FlyNap for 2–3 min (a few flies were anesthetized with FlyNap for 4–5 min as they were hard to narcotize). Then, the flies were fixed back down on a petri dish coated with medical petroleum jelly and artificial hemolymph was added (need to restore room temperature and pump oxygen for 15 min), Next, the head, ventral thorax and ventral abdominal cuticle were removed by special scissors and tweezers to expose the heart in the field of vision of a microscope. A EM-CCD high-speed camera was used to record the heartbeat of fruit flies (video at 130 fps, the 30 s), and HC Image software was used to record the cardiogram data. Semi-Automated Optical Heartbeat Analysis (SOHA) quantifies Heart Rate (HR), Heart Period (HP), Diastolic Intervals (DI), Systolic Intervals (SI), Arrhythmia Index (AI), Diastolic Diameter (DD), Systolic Diameter (SD), Fractional Shortening (FS), and Fibrillations (FL). Each group of samples was 20 ± 5.

### Negative Geotaxis Assay

According to the previous method (Birse and Bodmer, 2011). The climbing apparatus consisted of an 18-cm-long glass tube with an inner diameter of 2.8 cm (sponges were placed in the ends of the tube to prevent escape yet allow air exchange). Due to the instinctive negative geotaxis displayed by *Drosophila*, the flies climbed up the sides of the apparatus after being taped down at the bottom. Flies were allowed 8 s to climb after being taped down, and the climbing heights reached by the flies were calculated. A video camera was used to film the fly climbing during the whole process, the 4th, 5th, and 6th climbing images at the end of 8 s were intercepted, and the number of flies reaching the top was counted. Climbing index = number of in the uppermost area/total number.

### Triglyceride Assay

For quantification of TGs in whole flies, flies (15 per genotype) were weighed and homogenized in PBS containing 0.1% Triton-X100 in an amount (μl) that was 8 X the total weight of the flies (μg). Then, centrifugation was used and the supernatant was obtained. Fifty microliters of standard or sample was added to the appropriate wells. Blank wells had nothing added. One-hundred microliters of enzyme conjugate to was added standard wells and sample wells except for the blank well; they were covered with an adhesive strip and incubated for 60 min at 37°C. The microtiter plate was washed four times then, Substrate A (50 μl) and Substrate B (50 μl) were both added to each well, mixed gently, and incubated for 15 min at 37°C (while being protected from light). Following this, 50 μl stop solution was added to each well. The color in the wells should change from blue to yellow. If the well color was green or the color change does not appear uniform, the plate was gently tapped to ensure thorough mixing. The optical density (OD) was read at 450 nm using a microtiter plate reader within 15 min. Using the OD value of the measured standard product as the abscissa and the standard product’s concentration value as the ordinate, the standard curve was drawn to obtain the linear regression equation. The OD value of the sample was substituted into the equation to calculate the concentration of the sample.

### Oil Red O Staining

In order to quantify lipids, we stained the abdomen of *Drosophila* with Oil Red O. First, remove the intestines and fat bodies of fruit flies. Fix with 4% paraformaldehyde for 20 min and wash with PBS three times for 10 min each time. Filter the oil red O solution (the staining solution and distilled water are 3:2), then incubate at room temperature for 30 min and wash with PBS three times. The photos were taken under a Leica stereo microscope, and the images were processed using Adobe Photoshop.

### Statistical Analysis

Drawings were made using GraphPad Prism 6 software. Analyses were performed using the Statistical Package for the Social Sciences (SPSS) version 21.0 for Windows (SPSS Inc., Chicago, IL, United States). All data are presented as the mean ± SEM. A two-way ANOVA was used to identify differences among the NFD, NFD + E, HFD, and HFD + E groups of *Drosophila* with the same genetic background. Independent sample *t*-test was used to identify differences between W^1118^ > *apoLpp*^RNAi^ flies and Arm-Gal4 > *apoLpp*^RNAi^ or Hand-Gal4 > *apoLpp*^RNAi^ flies. A value of *p* < 0.05 was considered to be statistically significant.

## Results

### Cardiomyocyte-Derived *apoLpp* Plays an Important Role in the Regulation of Lipid Metabolism

**FIGURE 1:**
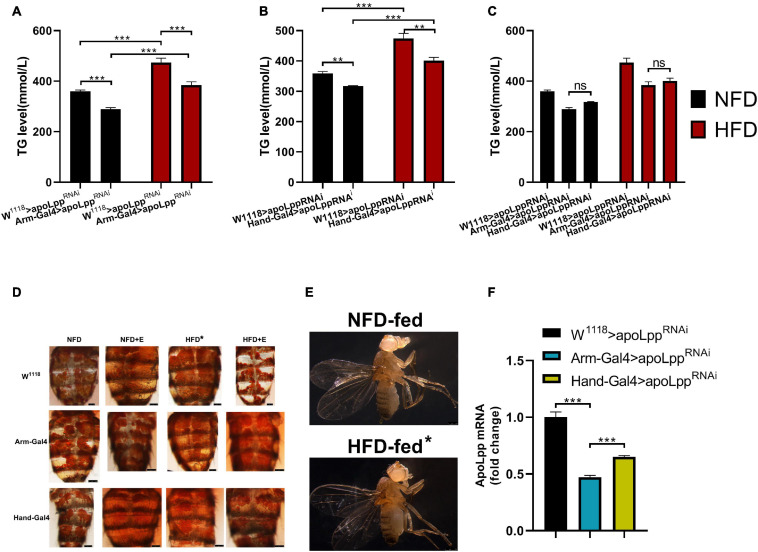
Whole-body triglyceride metabolism in *Drosophila*. **(A)** Effect of knockdown of whole-body apoLpp on whole-body TG levels under different dietary conditions. **(B)** Effect of specific knockdown of cardiac apoLpp on whole-body TG levels under different dietary conditions. **(C)** Differential effects of whole-body apoLpp knockdown vs. apoLpp cardiac-specific knockdown. **(D)** *Drosophila* abdominal ORO staining. **(E)** Lateral morphological photographs of *Drosophila*. When fed with HFD, the abdomen of *Drosophila* was significantly bulging and large in size. **(F)** The relative expression of apoLpp under normal dietary conditions with different knockdown effects. arm-Gal4 knockdown efficiency was 53%, while Hand-Gal4 knockdown efficiency was 35%. All samples were females. The whole-body triglyceride sample size was 15 and apoLpp mRNA sample volume of 60 hearts or 10 whole-body. Figures **(A,B)** use two-way ANOVA, and figures **(C,F)** use independent samples *t*-tests. *P < 0.05, **P < 0.01, ***P < 0.001.

To determine the important role of *Drosophila* cardiomyocyte-derived *apoLpp* in lipid metabolism, we used different degrees of knockdown to verify the differential contribution of *apoLpp* in the whole-body and the heart. We use the previous high-fat regimen to feed female flies on a NFD and HFD for 5 days (Diop et al., 2017). The measurement of the whole-body triglyceride (TG) level of flies showed that the whole-body TG level of flies fed on a HFD increased significantly (Figure 1A). Consistent with that, oil red O staining showed that HFD-fed flies had abdominal fat accumulation compared with the NFD group (Figure 1D). In addition, from the morphological photos of fruit flies, HFD-fed fruit flies showed a larger body size and a bulge in the abdomen. This showed that the high-fat regimen produced a diet-induced obese *Drosophila* model.

Next, we used two different Gal4s to drive knockdown of the whole-body and the heart, respectively. The measurement of whole-body TG levels showed that no matter under NFD or HFD conditions, compared with the W^1118^ > *apoLpp*^RNAi^ group, the Arm-Gal4 > *apoLpp*^RNAi^ group can reduce the whole-body TG level (Figure 1A). The results of the Hand-Gal4 > *apoLpp*^RNAi^ group were similar to those of the Arm-Gal4 > *apoLpp*^RNAi^ group (Figure 1B). Interestingly, although the Arm-Gal4 > *apoLpp*^RNAi^ group reduced the whole-body TG level under HFD conditions, it was still significantly higher than under NFD conditions (Figure 1A). The results of the Hand-Gal4 > *apoLpp*^RNAi^ group were similar to those of the Arm-Gal4 > *apoLpp*^RNAi^ group (Figure 1A). These results indicate that knockdown of *apoLpp* can to some extent resist the increase in HFD-induced whole-body TG levels, but it will not be like W^1118^ > *apoLpp*^RNAi^. In addition, we also compared these two Gal4 groups. The results showed that there was no significant difference in the knockdown effect of *apoLpp* between the Arm-Gal4 > *apoLpp*^RNAi^ and Hand-Gal4 > *apoLpp*^RNAi^ groups regardless of NFD or HFD conditions (Figure 1C). When we specifically knocked down cardiac *apoLpp*, it showed similar results as knocked down whole-body *apoLpp*. As a role of *apoLpp* in the control of lipid levels has previously been shown in *Drosophila* (Lee et al., 2017). Therefore, cardiac-specific knockdown of *apoLpp* plays an important role in controlling lipid levels. From the gene level, compared with the W^1118^ > *apoLpp*^RNAi^ group, the knockdown efficiency of the Arm-Gal4 > *apoLpp*^RNAi^ group was 53%, and the knockdown efficiency of the Hand-Gal4 > *apoLpp*^RNAi^ group was 35% (Figure 1F). In summary, these data illustrate the important role of *apoLpp* in controlling lipid levels, especially heart-derived *apoLpp*.

### Exercise-Training Rescues Low Exercise Capacity and Cardiac Insufficiency Caused by HFD

Exercise-training can improve exercise capacity and diastolic function (Nolte et al., 2014). There are many indicators to evaluate cardiac function. We used M-mode to quantify heart rate (HR), heart period (HP), systolic intervals (SI), and diastolic interval (DI), Systolic diameter (SD), Diastolic diameter (DD), Fractional shortening (FS), Fibrillations (FL), and Arrhythmia (AI). To explore the impact of exercise-training on the impaired exercise capacity and cardiac insufficiency caused by HFD, we made a climbing device using the negative geotaxis behavior (RING) of flies (Zheng et al., 2015).

**FIGURE 2:**
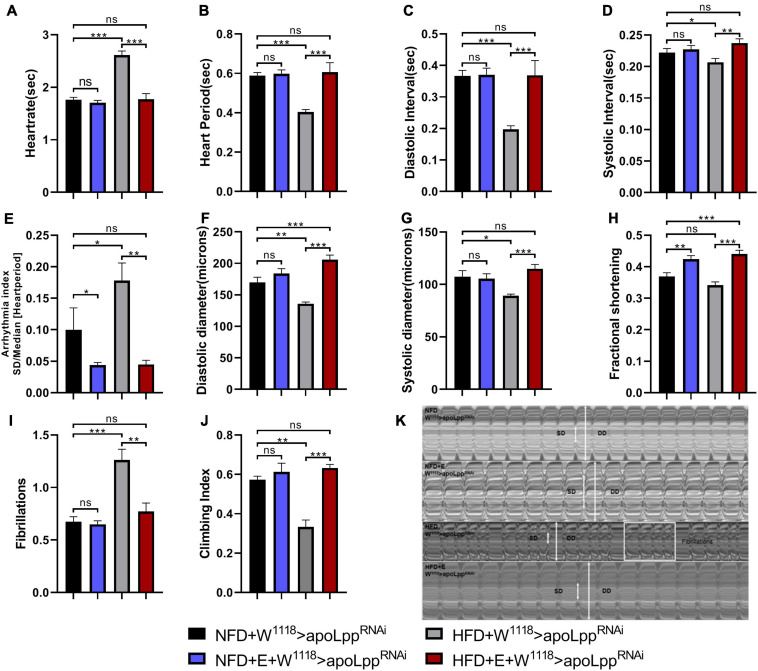
Changes in heart function, climbing ability, and TG levels of the W^1118^ > *apoLpp*^RNAi^ group under different conditions. **(A–I)** The M-mode indicators under different treatments are HR, HP, DI, SI, AI, DD, SD, FS, and FL. All flies were females, The sample sizes of the four groups NFD, NFD + E, HFD, and HFD + E are *N* = 16, 18, 21, and 15. **(J)** represents the climbing ability of fruit flies under different treatments. The value of the histogram is the climbing index. For calculation of the climbing index, see section “Materials and Methods.” All fruit flies were females, *N* = 50. **(K)** M-mode cardiogram of *Drosophila* under different treatments. The short arrow is the systolic diameter, the long arrow is the diastolic diameter, and the rectangle is the fibrillary tremor. The interception of M-mode cardiogram for each group is 10 s. All *P*-values are derived from two-way ANOVA. LSD was used for *post-hoc* testing. *P < 0.05, **P < 0.01, ***P < 0.001.

Next, we performed exercise-training for 5 days at the same time as HFD feeding. M-mode results showed that, compared with NFD, W^1118^ > *apoLpp*^RNAi^ under HFD conditions significantly increased HR, AI, and FL (Figures 2A,E,I). HP, DI, SI, DD, and SD decreased significantly (Figures 2B–D,E,F). FS showed no significant difference. These data indicate that under HFD conditions, the heartbeat of fruit flies is accelerated, accompanied by arrhythmia and fibrillation (Figure 2K), but the shortening score does not change significantly. This makes it easy for us to think of Arrhythmia-induced cardiomyopathy (Sossalla and Vollmann, 2018). This indicates that the heart function of flies is impaired under HFD. Comparing NFD + W^1118^ > *apoLpp*^RNAi^ and NFD + E + W^1118^ > *apoLpp*^RNAi^ group, there were no significant differences in HR, HP, DI, SI, DD, SD, and FL in exercise-training flies (Figures 2A–D,F,G,I), but AI significantly decreased and FS increased significantly (Figures 2E,H). This shows that under NFD conditions, the contribution of exercise-training to heart function is at least partially augmented. Compared with the HFD + W^1118^ > *apoLpp*^RNAi^ group, the HFD + E + W^1118^ > *apoLpp*^RNAi^ group had significantly lower HR, AI, and FL (Figures 2A,E,I), and significantly increased HP, DI, SI, DD, SD, and FS (Figures 2B–D,F–H). In addition, NFD + E + W^1118^ > *apoLpp*^RNAi^ and HFD + E + W^1118^ > *apoLpp*^RNAi^ group have no significant difference in cardiac function indicators (Figures 2A–I). In summary, especially for HFD-induced heart damage, these data demonstrated that *Drosophila* exercise-training improved heart function. When overeating high-fat food, flies can reduce the risk of cardiac function damage through exercise-training and increase the heart Fractional shortening and strengthen the heart pumping function.

We tested the climbing ability and TG levels of flies. The results showed that the climbing index of flies was very different under different dietary conditions. HFD feeding caused a significant decrease in the climbing index of flies and increased the level of TG in the whole-body. *Drosophila* with exercise-training can slightly improve climbing ability during NFD (statistically no significant difference), and reduce the whole-body TG level (Figure 2J and Supplementary Figure 1). However, when fed with HFD, exercise-training can resist the low climbing ability caused by HFD, and it can also resist the increase of TG levels in the whole-body (Figure 2J and Supplementary Figure 1). These results indicate that exercise-training can regulate the lipid level of the whole-body and reduce the systemic TG so that the heart is protected from functional impairment caused by a HFD. It can also improve climbing ability to a level slightly higher than normal. Simultaneously, a decrease in heart rate and the increase in systolic diameter suggest that exercise-training has a beneficial effect on the heart’s pumping function. This explains to a certain extent the possibility of exercise-training to improve heart function.

### Exercise-Training Combined With the Inhibition of Whole-Body *apoLpp* to Attenuate HFD-Induced Impairment of Heart Function and Low Exercise Capacity

**FIGURE 3:**
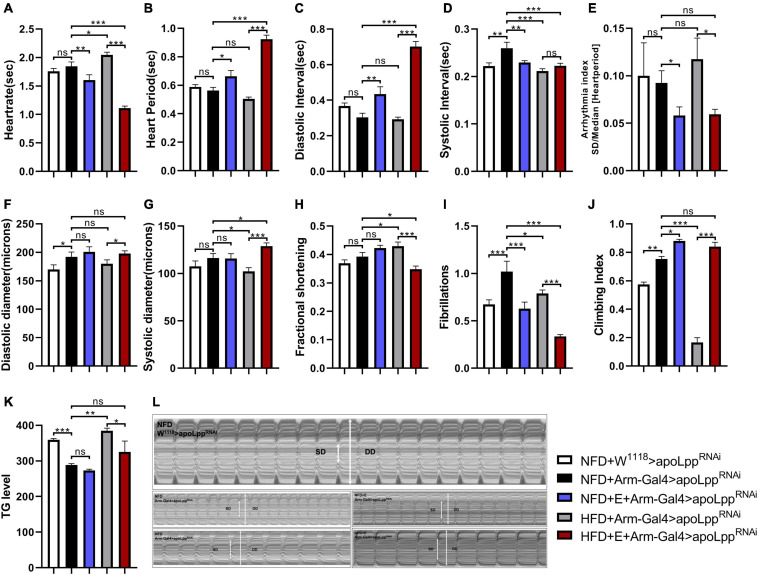
The effect of whole-body inhibition of *apoLpp* on the heart function, exercise capacity, and TG levels of each group of *Drosophila*. **(A–I)** Under different treatments, the M-mode indicators are HR, HP, DI, SI, AI, DD, SD, FS, and FL. All flies were females, The sample sizes, NFD + w^1118^ > *apoLpp*^RNAi^, NFD + Arm-Gal4 > *apoLpp*^RNAi^, NFD + E + Arm-Gal4 > *apoLpp*^RNAi^, HFD + Arm-Gal4 > *apoLpp*^RNAi^, and HFD + E + Arm-Gal4 > *apoLpp*^RNAi^ are *N* = 16, 18, 14, 17, and 22. **(J)** Represents the locomotor ability of flies under different treatments. The value of the histogram is the climbing index of the flies. For the calculation of the climbing index, see section “Materials and Methods.” All flies were females, *N* = 50. **(K)** Whole-body TG levels in flies under different treatments. The bar graph value is the original OD value/fly’s body weight. All samples were females, *N* = 5, and the measurement was repeated three times. **(L)** M-mode cardiogram of *Drosophila* under different treatments. The short arrow is the systolic diameter, the long arrow is the diastolic diameter, and the rectangle is the fibrillary tremor. The interception of M-mode cardiogram for each group was 10 s. Independent sample *t*-test was used to identify differences between NFD + w^1118^ > *apoLpp*^RNAi^ flies and NFD + Arm-Gal4 > *apoLpp*^RNAi^. A two-way ANOVA was used to identify differences among the NFD, NFD + E, HFD, and HFD + E groups in Arm-Gal4 > *apoLpp*^RNAi^ flies. *P < 0.05, **P < 0.01, ***P < 0.001.

When the NFD + Arm-Gal4 > *apoLpp*^RNAi^ group was compared with the control group NFD + W^1118^ > *apoLpp*^RNAi^, the whole-body KD of *apoLpp* significantly increased SI, DD, and FL (Figures 3D,F,I). It suggests that the whole-body KD of *apoLpp* increases the incidence of fly’s fibrotic tremors. In addition, the whole-body KD of *apoLpp* also increased the climbing index (Figure 3J) and lowered the whole-body TG level (Figure 3K). This suggests that the KD of the whole-body *apoLpp* can reduce the whole-body TG level of flies and improve the climbing ability. However, it is interesting that although the knockdown of whole-body *apoLpp* can improve climbing ability, it also increases the incidence of cardiac fibrillation, which may be caused by excessive loss of *apoLpp*.

In addition, although previous studies have confirmed that HFD can cause impaired heart function in flies, it is unclear whether knockdown of *apoLpp* can affect various indicators of heart function. In order to figure this out, we knocked down the whole-body apoLpp of HFD-fed flies. The results showed that compared with the NFD + Arm-Gal4 > *apoLpp*^RNAi^ group, HFD + Arm-Gal4 > *apoLpp*^RNAi^ flies HR, FS significantly increased (Figures 3A,H), but not like HFD + W^1118^ > *apoLpp*^RNAi^ group is as high. This suggests that the knockdown of HFD combined with the whole-body *apoLpp* can to some extent resist the increase in heart rate and contractile dysfunction induced by HFD. We also found that HFD combined with whole-body *apoLpp* knockdown reduced SI, SD and FL (Figures 3D,J,I). No difference between HP and DI (Figures 3B,C). It is suggested that HFD stimulation did not increase the incidence of fibrillation (Figure 3L), even lower than that under NFD conditions. In addition, HFD combined with the KD of the whole-body *apoLpp* significantly reduced the flies climbing index (Figure 3J) and increased the whole-body TG level (Figure 3K). This suggests that the KD of *apoLpp* is not enough to resist the low climbing ability caused by HFD, because the KD of the whole-body *apoLpp* under HFD conditions does not save the climbing ability of flies as it did during NFD. These results prove that under NFD conditions, the whole-body KD of *apoLpp* improves the climbing ability of flies, but it increases the risk of fibrillation. Under the condition of HFD, the whole-body KD of *apoLpp* can resist HFD-induced cardiac function damage to a certain extent, but it is not enough to resist HFD-induced low exercise capacity.

We know that exercise-training is an effective way to improve heart function and exercise capacity, but it is still unclear about the relationship between exercise-training and the KD of the whole-body *apoLpp* in the heart function and exercise capacity. In order to figure this out, we conducted exercise-training for flies in the NFD + Arm-Gal4 > *apoLpp*^RNAi^ and HFD + Arm-Gal4 > *apoLpp*^RNAi^ groups. The data showed that fruit flies after exercise-training significantly reduced HR, AI, and FL (Figures 3A,E,I). Interestingly, under NFD conditions, exercise-training did not significantly change the DD, SD, and FS (Figures 3F–H) of flies. Under HFD conditions, DD and SD (Figures 3F,G) of flies after exercise-training increased significantly, while FS (Figure 3F) decreased significantly. This reminds us of dilated cardiomyopathy. In addition, *Drosophila* climbing index and whole-body TG level (Figures 3J,K) show that exercise-training significantly improves climbing ability and can reduce the increase in whole-body TG level caused by HFD feeding. These results prove that exercise-training can improve climbing ability, resist the increase in heart rate caused by HFD, and reduce the occurrence of arrhythmia and fibrillation. In addition, under HFD conditions, exercise-training may also lead to the occurrence of dilated cardiomyopathy. This may be caused by the superposition of the whole-body KD of *apoLpp* and exercise-training.

### Exercise-Training Combined With the Inhibition of Cardiomyocyte-Derived *apoLpp* to Improve HFD-Induced Impairment of Heart Function and Low Exercise Capacity

**FIGURE 4:**
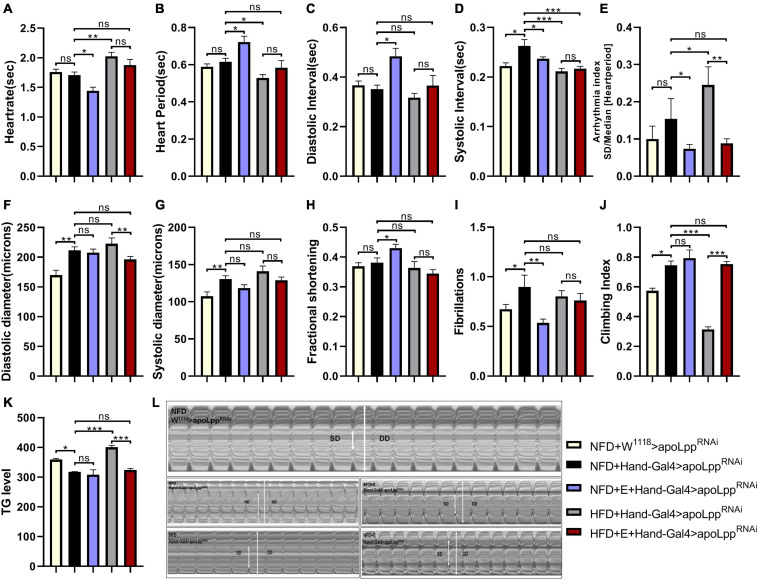
Effect of cardiomyocyte-specific inhibition *apoLpp* on heart function, exercise capacity, and TG levels of each fly group. **(A–I)** Under different treatments, the M-mode indicators are HR, HP, DI, SI, AI, DD, SD, FS, and FL. All fruit flies were females, The sample sizes, NFD + w^1118^ > *apoLpp*^RNAi^, HFD + w^1118^ > *apoLpp*^RNAi^, NFD + Hand-Gal4 > *apoLpp*^RNAi^, and HFD + Hand-Gal4 > *apoLpp*^RNAi^ are *N* = 16, 21, 19, and 16. **(J)** represents the locomotor ability of flies under different treatments. The value of the histogram is the climbing index of flies. For the calculation of the climbing index, see section “Materials and Methods.” All flies were females, *N* = 50. **(K)** Whole-body TG levels in flies under different treatments. The bar graph value is the original OD value/fly’s body weight. All samples were females, *N* = 5, and the measurement was repeated three times. **(L)** M-mode cardiogram of *Drosophila* under different treatments. The short arrow is the systolic diameter, the long arrow is the diastolic diameter, and the rectangle is the fibrillary tremor. The interception of M-mode cardiogram for each group was 10 s. Independent sample *t*-test was used to identify differences between NFD + w^1118^ > *apoLpp*^RNAi^ flies and NFD + Hand-Gal4 > *apoLpp*^RNAi^. A two-way ANOVA was used to identify differences among the NFD, NFD + E, HFD, and HFD + E groups in Hand-Gal4 > *apoLpp*^RNAi^ flies. *P < 0.05, **P < 0.01, ***P < 0.001.

Using myocardial-specific Hand-Gal4 to drive the KD of *apoLpp* in the heart, we found that, compared to the NFD + W^1118^ > *apoLpp*^RNAi^ group, the KD of *apoLpp* of cardiomyocytes caused a significant increase in SI, DD, SD, and FL (Figures 4D,F,G,I), there is no significant difference in the rest of the heart function indicators (Figure 4C). The KD of *apoLpp* of cardiomyocytes also significantly reduced TG level in the whole-body of flies and improved climbing ability (Figures 4J,K). We found that although the KD of *apoLpp* of cardiomyocytes can increase the diastolic and systolic diameters, there is no change in the shortening fraction, which suggests that the heart pumping function is weakened. In addition, it is accompanied by the occurrence of fibrillation (Figure 4L), which indicates that the KD of the cardiomyocyte *apoLpp* has certain side effects on the heart function of flies, but it can improve the climbing ability of flies. To determine whether *apoLpp* of cardiomyocytes-derived can resist HFD-induced damage, we gave flies in the Hand-Gal4 > *apoLpp*^RNAi^ group a HFD. We found that compared with the NFD + Hand-Gal4 > *apoLpp*^RNAi^ group, HFD feeding caused a significant increase in HR and AI (Figures 4A,E), a significant decrease in HP and SI (Figures 4B,D), and no significant difference in other cardiac function indicators. Although the heart rate and arrhythmia index have improved, they are not as high as those in the W^1118^ group fed by HFD. This indicates that the KD of *apoLpp* of cardiomyocytes is at least partially resistant to HFD-induced cardiac function damage. In addition, we also found that in the HFD + Hand-Gal4 > *apoLpp*^RNAi^ group, the whole-body TG level of flies was increased and the climbing index was significantly decreased (Figures 4K,J), which suggested that the KD of *apoLpp* of cardiomyocytes was not enough to resist the increase of TG and the low climbing ability induced by HFD. Interestingly, the increase in whole-body TG levels was not as high as that of flies in the W^1118^ group, which indicates that the KD of *apoLpp* in cardiomyocytes can partially resist the increase in systemic TG levels induced by HFD. The climbing index has been at a low level, indicating that the KD effect of *apoLpp* in cardiomyocytes has no significant effect on the low climbing ability induced by HFD.

Although it has been established that exercise-training can alleviate the heart function impairment and exercise capacity induced by flies HFD feeding, it is not clear whether exercise-training and the knockdown of *apoLpp* of cardiomyocytes have an additive effect. In order to figure this out, we applied exercise-training to flies in the Hand-Gal4 > *apoLpp*^RNAi^ group. We found that exercise-training significantly reduced HR, AI, and FL under NFD conditions while increasing FS (Figures 4A,E,I,H). This shows that exercise-raining combined with *apoLpp* knockdown of cardiomyocytes can further reduce the heart rate and increase the shortening score, showing bradycardia. In addition, the reduced incidence of arrhythmia and fibrillation suggests that exercise-training can alleviate the damage caused by knockdown of *apoLpp* of cardiomyocytes. Under HFD conditions, exercise-trained flies significantly reduced AI and whole-body TG levels and significantly increased climbing index (Figures 4E,K,J). This shows that exercise combined with the KD of cardiomyocytes *apoLpp* can further reduce arrhythmia, reduce the level of TG in the whole-body, and improve the climbing ability. In general, the effects of exercise-training and cardiomyocyte *apoLpp* knockdown are superimposed, and they are mainly manifested in the heart rate, arrhythmia index, fibrillation and shortening score. In addition, exercise-training combined with the knockdown of *apoLpp* of cardiomyocytes can further reduce the occurrence of arrhythmia and resist the elevated levels of TG and low climbing ability induced by HFD.

### Exercise-Training Under Different Dietary Conditions Changed the Expression Level of *apoLpp*

Exercise is a complex process that affects most aspects of the cardiovascular system (Roh et al., 2016). It can reduce cardiovascular risk factors, including hyperlipidemia (Mannu et al., 2013), hypertension (Pal et al., 2013), diabetes (LeBrasseur et al., 2011), and can also benefit patients with cardiovascular disease. It is similar to some drug interventions (Naci and Ioannidis, 2013). Novel areas for exercise-training interventions include HF with preserved ejection fraction (HFpEF), pulmonary hypertension, and valvular heart disease (Gielen et al., 2015). In HFpEF, randomized studies indicate a lusitropic effect of training on left ventricular diastolic function associated with symptomatic improvement of exercise capacity (Gielen et al., 2015). To better understand the relationship between exercise-training and *apoLpp*, we tested the expression level of *apoLpp* mRNA in flies.

**FIGURE 5:**
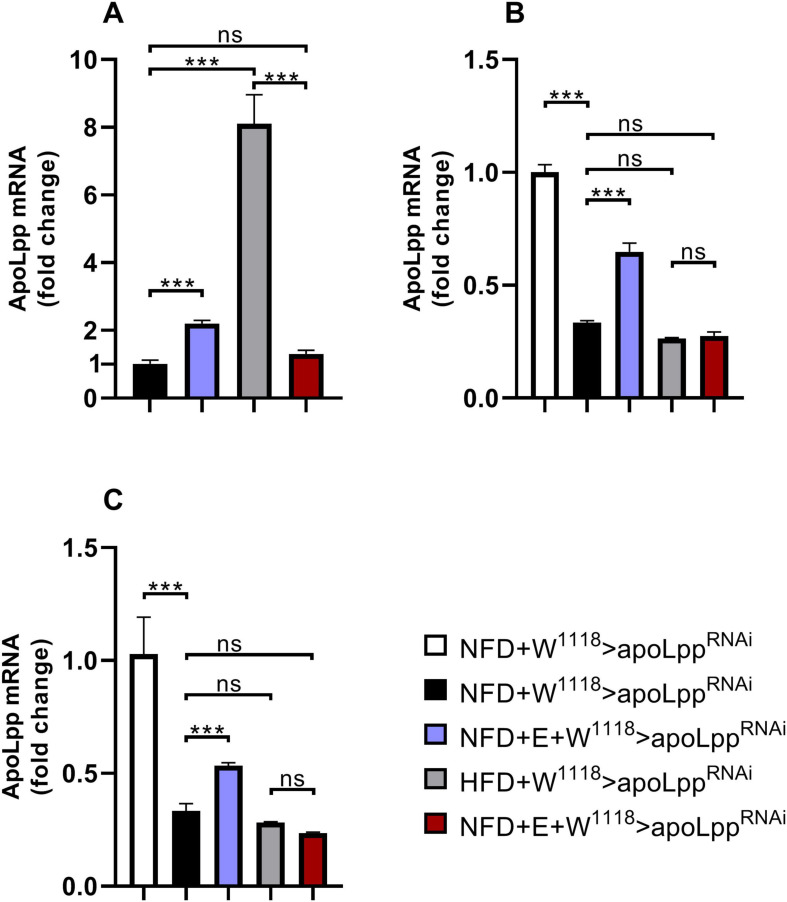
*Drosophila* heart and whole-body *apoLpp* mRNA relative expression level. **(A)** W^1118^ group flies heart *apoLpp* expression level. **(B)** Arm group flies heart *apoLpp* expression level. **(C)** Hand group flies heart *apoLpp* expression level. All flies were females, and the sample size of each group was 60 hearts or 10 whole-body. GAPDH was used to normalize the values. Independent sample *t*-test was used to identify differences between W^1118^ > *apoLpp*^RNAi^ flies and Arm-Gal4 > *apoLpp*^RNAi^ or Hand-Gal4 > *apoLpp*^RNAi^ flies. A two-way ANOVA was used to identify differences among the NFD, NFD + E, HFD, and HFD + E groups in Arm-Gal4 > *apoLpp*^RNAi^ or Hand-Gal4 > *apoLpp*^RNAi^ flies. *P < 0.05, **P < 0.01, ***P < 0.001.

We found that under the conditions of NFD, W^1118^ groups, Arm group and Hand group of flies were all increased in the expression of *apoLpp* after exercise-training (Figures 5A–C). This may be due to the increase in lipid metabolic cycles in order to meet the energy supply required for training in normal diet. Under HFD conditions, the expression level of *Drosophila apoLpp* was significantly increased, significantly decreased after exercise-training, and there was no significant difference with the control group (Figure 5A). This indicates that exercise-training can effectively reduce the expression level of *apoLpp*. The *apoLpp* expression level in the Arm group and the Hand group did not rise significantly, which means that the Arm group and the Hand group can effectively drive the heart and whole-body *apoLpp* knockdown. Interestingly, under HFD conditions, exercise-training did not significantly affect the expression of Arm group flies *apoLpp*, but significantly reduced the *apoLpp* expression levels of the Hand group flies (Figures 5B,C). This indicates that the exercise-training has superposed on the suppression of *apoLpp*.

## Discussion

Obesity caused by HFD affects heart function and exercise capacity. Obesity is an important cause of diabetes, cardiovascular disease, and low exercise capacity. Obesity caused by a HFD diet dramatically affects the incidence of cardiovascular disease. We discussed possible uses of exercise-training to reduce heart function impairment and low exercise capacity caused by HFD. Our main findings are: (1) Under the conditions of high-fat feeding, the low expression of *apolpp* helps alleviate HFD-induced heart functional damage. (2) Exercise-training combined with *apoLpp* low expression can resist HFD-induced heart functional damage and low climbing ability. These results clarify the previously unknown relationship between *apoLpp* and cardiac function and exercise capacity and suggest possible ways for *apoLpp* in an exercise-regulated heart to improve cardiac function and exercise capacity.

*Drosophila* is a good model organism for studying heart function. Its heart is a tubular structure that pumps hemolymph in an open circulatory system. Development of the heart occurs through evolutionarily conserved molecular mechanisms. Because of its simple structure and the availability of powerful genetic tools, the *Drosophila* heart has become a model system for studying the fundamental genetic and molecular mechanisms of cardiac development, function and aging (Ocorr et al., 2014). Diet, physical exercise, and environmental factors are related to diabetes and cardiovascular disease (Howard et al., 2006; Morera et al., 2019). Exercise-training causes profound beneficial changes in skeletal muscle and heart function (Piazza et al., 2009). However, the molecular mechanisms by which exercise-training regulates heart function are poorly understood. Therefore, we used this simple biological model to illustrate the internal connection between exercise-training and cardiac function. We revealed the role of *apoLpp* in cardiac function and exercise capacity, as well as the regulatory effect of exercise-training on *apoLpp* in cardiomyocytes.

In humans, apoB is important in preventing coronary heart disease (Peloso et al., 2019) and is a non-traditional lipid biomarker (Muscella et al., 2020). The apoB48 assembles chylomicrons and apoB100 assembles very low-density lipoprotein (VLDL) (Sirwi and Hussain, 2018). Triglycerides are routinely used to measure VLDL. An increase in triglyceride levels increases the risk of cardiovascular disease and a decrease in plasma triglycerides reduces the risk of cardiovascular disease (Sniderman et al., 2018). In this study we found that the systemic triglyceride levels are elevated under HFD conditions, and the effect of *apoLpp* on heart function is mainly to resist the increase in heart rate and the occurrence of fibrillation. The fibrillation observed in flies is similar to human atrial fibrillation (Wiersma et al., 2019). It is the most common persistent arrhythmia (Vaquero et al., 2008), and coronary heart disease (CHD) and atrial fibrillation (AF) usually co-occur (Thompson and Morton, 2016). Our results demonstrated that exercise-training changes the expression of *apoLpp* in *Drosophila* cardiomyocytes. A reduction of apoB after exercise-training has also been found in humans (Holme et al., 2007). For example, exercise-training can improve blood lipid status and affect lipoprotein levels (Durstine and Haskell, 1994). After exercise-training, HDL-cholesterol (HDL-C) and apolipoprotein (apo) A-I increased, while triglycerides and apo B decreased (Thompson et al., 1997). Fitness and physical activity had inverse associations with incident cardiovascular disease and also in individuals with elevated genetic risk for these diseases. In particular, a high level of cardiopulmonary adaptability can significantly reduce the risk of coronary heart disease (Tikkanen et al., 2018). However, in rodents, apolipoprotein B is important in reducing liver lipid accumulation during the progression of obesity (Miotto et al., 2017). Exercise-training reduces the expression of MTP and apoB (Lira et al., 2008), and the serum concentrations of triglycerides, free and esterified cholesterol and apolipoprotein B are significantly reduced (Shepherd et al., 1985). Our results show that in *Drosophila*, the effects of exercise-training on lipoproteins are similar to humans, especially in terms of heart function.

In flies from the W^1118^ group with NFD-fed, exercise-training was able to increase cardiac *apoLpp* expression and decrease whole-body triglyceride levels. M-mode showed that the incidence of arrhythmias was decreased and shortening fraction was increased in exercise-trained flies, suggesting enhanced cardiac function. Under HFD feeding, *apoLpp* derived from cardiomyocytes was upregulated about eightfold and *Drosophila* heart function was impaired and abdominal fat accumulation (Figures 1D,E) with a significant reduction in climbing ability. In contrast, after a week of exercise-training *apoLpp* expression was significantly decreased, exercise capacity was enhanced, and *Drosophila* cardiac function recovered and no obvious lipid accumulation in the abdomen (Figure 1D), even exhibiting a higher shortening fraction than normal controls and a lower incidence of arrhythmias. In addition, the triglyceride data also illustrated that systemic TG levels were significantly elevated in flies when HFD feeding and significantly decreased after the imposition of a week of exercise-training. That is, HFD feeding resulted in a dramatic increase in cardiogenic *apoLpp* mRNA, which may have secreted more Apolipoprotein to bind circulating neutral lipids, resulting in significantly higher whole-body TG levels, and exercise-training resisted this effect of HFD-induced *apoLpp* overexpression, results that are strikingly similar compared to humans.

The data also showed that flies in the Arm group under NFD conditions, exercise-training increased whole-body *apoLpp* expression levels, but whole-body triglyceride levels remained unchanged. In addition, flies showed increased exercise capacity and improved cardiac function, mainly in the form of arrhythmias and reduced incidence of fibrillation. In contrast, the effect of exercise-training on whole-body *apoLpp* was not significant under HFD conditions, and there were no significant changes in whole-body triglyceride levels or climbing ability, and abdominal fat does not accumulate (Figure 1D) which further improved cardiac function in flies, suggesting that exercise-training combined with knockdown of whole-body *apoLpp* could resist HFD-induced cardiac impairment and low climbing ability. The exercise-training to increase cardiac *apoLpp* expression levels without significant changes in whole-body triglyceride levels. Suggests that exercise-training increased lipoprotein activity and increased neutral lipid translocation and utilization to adapt to the changing environment of the body, resulting in increased *apoLpp* expression but no significant differences in whole-body triglyceride levels, which is consistent with the results of the Arm group under NFD conditions. In addition, the climbing ability of flies did not change significantly compared to its control group but was significantly higher than that of flies in the W^1118^ group, a result that may be due to the knockdown effect that itself increased climbing ability without the superimposed effect of the imposed exercise-training. In contrast, under HFD conditions, flies in the Hand group showed no significant changes in cardiac *apoLpp* expression levels, systemic triglyceride levels, or climbing ability after exercise-training, there is no obvious fat accumulation (Figure 1D). In addition, flies heart function was similar to Hand control group. This suggests that specific knockdown of cardiac *apoLpp* combined with exercise-training can effectively reduce the impairment of cardiac function and low climbing ability caused by HFD feeding. In short, a HFD is harmful to heart function and exercise capacity. It shows the accumulation of apoLpp in the body. Exercise-training and limit apoLpp expression can alleviate this situation, but considering that apolipoprotein B can shuttle in the body, it may also actively excrete lipids out of the organs during a HFD, thereby protecting the organs from lipotoxicity damage. Therefore, the effect of high-fat feeding combined with exercise-training in reducing apolipoprotein B is not absolute. This subtle relationship between them is worth studying.

## Conclusion

In conclusion, our results demonstrate the important role of *Drosophila* Apo B for cardiac function and exercise capacity under different dietary conditions. In addition, exercise-training to regulate combined *apoLpp* low expression against HFD-induced impairment of cardiac function and low exercise capacity.

## Data Availability Statement

The raw data supporting the conclusions of this article will be made available by the authors, without undue reservation.

## Ethics Statement

The animal study was reviewed and approved by the Ethics Committee of Hunan Normal University.

## Author Contributions

MD and LZ conceived and designed the experiments and wrote the manuscript. MD, QL, WW, and MZ collected the samples. MD, WW, WP, and MZ performed the experiments. MD analyzed the data. All authors have read and approved the final manuscript.

## Conflict of Interest

The authors declare that the research was conducted in the absence of any commercial or financial relationships that could be construed as a potential conflict of interest.
