# Supplementary figures and images for: Exercise-Training Regulates Apolipoprotein B in Drosophila to Improve HFD-Mediated Cardiac Function Damage and Low Exercise Capacity
Source: Front Physiol. 2021 Jul 7;12:650959. doi: 10.3389/fphys.2021.650959 (PMC8294119; doi:10.3389/fphys.2021.650959)

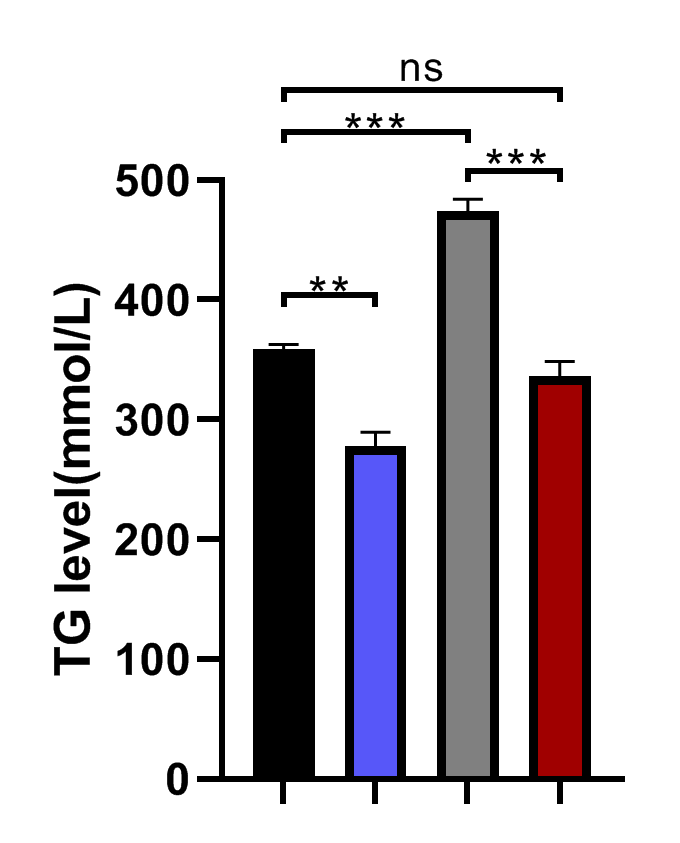

Supplement: Supplementary file 1 [file Image_1.TIF]
